# Inhibitors of Human ABCG2: From Technical Background to Recent Updates With Clinical Implications

**DOI:** 10.3389/fphar.2019.00208

**Published:** 2019-03-05

**Authors:** Yu Toyoda, Tappei Takada, Hiroshi Suzuki

**Affiliations:** Department of Pharmacy, The University of Tokyo Hospital, Faculty of Medicine, The University of Tokyo, Tokyo, Japan

**Keywords:** BCRP, cancer chemotherapy, drug repurposing, febuxostat, Ko143, multidrug resistance, tumor lysis syndrome, vesicle transport

## Abstract

The ATP-binding cassette transporter G2 (ABCG2; also known as breast cancer resistance protein, BCRP) has been suggested to be involved in clinical multidrug resistance (MDR) in cancer like other ABC transporters such as ABCB1 (*P*-glycoprotein). As an efflux pump exhibiting a broad substrate specificity localized on cellular plasma membrane, ABCG2 excretes a variety of endogenous and exogenous substrates including chemotherapeutic agents, such as mitoxantrone and several tyrosine kinase inhibitors. Moreover, in the normal tissues, ABCG2 is expressed on the apical membranes and plays a pivotal role in tissue protection against various xenobiotics. For this reason, ABCG2 is recognized to be an important determinant of the pharmacokinetic characteristics of its substrate drugs. Although the clinical relevance of reversing the ABCG2-mediated MDR has been inconclusive, an appropriate modulation of ABCG2 function during chemotherapy should logically enhance the efficacy of anti-cancer agents by overcoming the MDR phenotype and/or improving their pharmacokinetics. To confirm this possibility, considerable efforts have been devoted to developing ABCG2 inhibitors, although there is no clinically available substance for this purpose. As a clue for addressing this issue, this mini-review provides integrated information covering the technical backgrounds necessary to evaluate the ABCG2 inhibitory effects on the target compounds and a current update on the ABCG2 inhibitors. This essentially includes our recent findings, as we serendipitously identified febuxostat, a well-used agent for hyperuricemia as a strong ABCG2 inhibitor, that possesses some promising potentials. We hope that an overview described here will add value to further studies involving in the multidrug transporters.

## Introduction

Two decades ago, the ABC transporter G2 (ABCG2) was discovered in drug-resistant cancer cells and human placenta (Allikmets et al., 1998; Doyle et al., 1998; Miyake et al., 1999). Thereafter, many studies were conducted to determine the role of ABCG2 in developing MDR in cancer. Moreover, in the first decade, *in vivo* studies using *Abcg2* knockout mice (Jonker et al., 2002) coupled with biochemical characterizations revealed the importance of ABCG2 in the biological defense mechanisms against xenobiotics (Vlaming et al., 2009). Indeed, ABCG2—a 655-amino acid protein working as a homodimer on cellular plasma membranes (Robey et al., 2009)—is expressed not only in cancer cells but also in several normal tissues, such as brush border membranes of epithelium in the intestine and of proximal tubules in the kidney, bile canalicular membranes of the hepatocytes in the liver, luminal membranes of the mammary gland epithelium, and blood-facing membranes of the endothelial cells forming the BBB. In these tissues ABCG2 plays a pivotal role in the extrusion of various endogenous and exogenous substrates including drugs (Mizuno et al., 2004, 2007; Adachi et al., 2005; Hirano et al., 2005; Jonker et al., 2005; Ando et al., 2007). Hence, this transporter is recognized as an important determinant of the pharmacokinetic characteristics profiles of various drugs (Giacomini et al., 2010).

In the next decade, after identifying ABCG2 as a physiologically important urate transporter, a positive relationship between ABCG2 dysfunction and increased risk of human diseases, such as gout and hyperuricemia was revealed (Matsuo et al., 2009; Woodward et al., 2009; Ichida et al., 2012; Higashino et al., 2017). In addition to the sulfate conjugates of endogenous steroids (Suzuki et al., 2003) and porphyrins (Zhou et al., 2005; Robey et al., 2009), phytoestrogen sulfate conjugates (van de Wetering and Sapthu, 2012) and a uremic toxin indoxyl sulfate (Takada et al., 2018) were added in the growing list of ABCG2 substrates. Contrary to these advances in understanding the pathophysiological importance of ABCG2, the clinical relevance of reversing ABCG2-mediated MDR has been inconclusive (Robey et al., 2018).

ABCG2 overexpression can render the cancer cells resistant to the ABCG2 substrate chemotherapy agents, such as mitoxantrone, doxorubicin, SN-38, and several TKIs. To the best of our knowledge, no published clinical trial has ever succeeded in reversing the ABCG2-mediated MDR. This is because, despite a lot of efforts in ABCG2 inhibitor development, chemical knock-out/down of ABCG2 in clinical situations has not been achieved yet due to the lack of an appropriate candidate molecule. We herein describe some well-used experimental systems to evaluate the ABCG2 inhibitory activity, followed by a recent update on the ABCG2 inhibitors that includes a potent substance, febuxostat.

## Technical Background for Functional Validation

**FIGURE 1:**
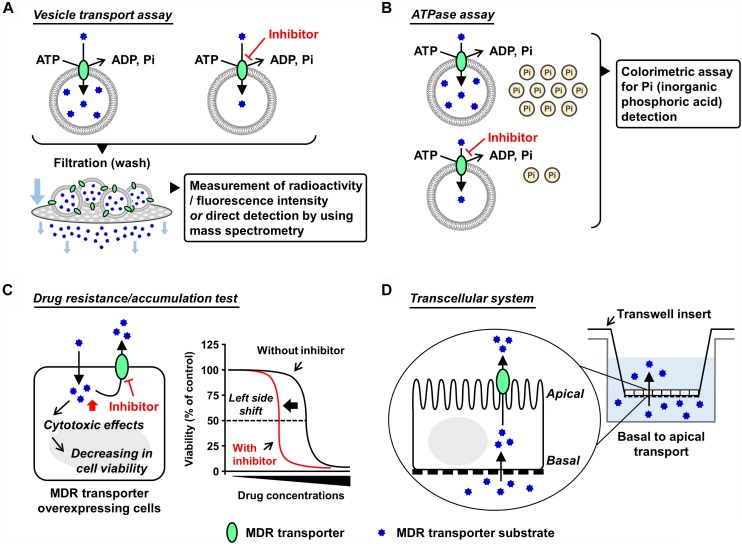
Schematic illustrations of each *in vitro* assay. Generally used *in vitro* models which are classified into membrane-based systems and cell-based systems (Hegedus et al., 2009) are shown. In the former systems, investigators can use culture cell-derived plasma membrane vesicles or reconstituted proteoliposomes as described in the main text. In the latter systems, aside from a couple of exceptions using *Xenopus laevis* oocytes (Nakanishi et al., 2003; Woodward et al., 2009), mammalian cells expressing target ABC protein are generally used. **(A,B)** Plasma membrane vesicle- or proteoliposome-based methods: vesicle transport assay **(A)** and ATPase assay **(B)**. Both plasma membrane vesicles and reconstituted proteoliposomes are applicable to the vesicle transport assay and the ATPase assay. Of note, the final step of the vesicle preparation—gentle homogenization of isolated membrane fraction—is empirically important for the formation of inside-out plasma membrane vesicles, whose outer faces are the cytoplasmic aspects of the parent membranes. Although the resulting plasma membrane vesicles are the mixture of inside-out and right-side-out components, without any separation of the right-side-out vesicles they are generally stored at –80°C and subjected to further assays. This is because that in these *in vitro* assays, only ABC proteins embedded in the inside-out vesicles have their ABCs outside of the vesicles and can use ATP in the reaction mixture for their transport function. In other words, the ABC proteins in the right-side-out vesicles cannot work due to an inaccessibility of the ABCs with ATP. Additionally, ABCG2-enriched plasma membrane vesicles are used for a biochemical analysis to study interactions of candidate chemicals with ABCG2 at the substrate-binding sites, known as the photoaffinity labeling of ABCG2 with [^125^I]-iodoarylazido-prazosin (Shukla et al., 2006). **(C,D)** Cell-based methods: drug resistance/accumulation test **(C)** and transcellular system **(D)**. MDR, multidrug resistance.

Various experimental models are available to examine the functions of the ABC transporters. Mainly focusing on ABCG2, with a current update this section introduces some *in vitro* and *in vivo* models that have been used in ABC transporter field. Broadly, the *in vitro* models are classified into two types, namely membrane-based systems and cell-based systems (Figure 1).

### Plasma Membrane Vesicle-Based Methods

#### Preparation of Plasma Membrane Vesicles

In mammals, most of the ABC transporters are membrane proteins and work as an efflux pump involved in the transport of its substrates from the cytosol, either to the extracellular space or into organelles by an ATP-dependent manner. Therefore, isolation of the target ABC protein-enriched cell membrane is the first step for biochemical analyses. For ABCG2, sucrose density gradient ultracentrifugation for the isolation of plasma membrane fraction is generally employed to prepare plasma membrane vesicles from ABCG2-expressing cells (related notes are inscribed in the legend of Figure 1). For this purpose, not only mammalian cells but also insect cells [e.g., baculovirus-infected Sf9 cells (Saito et al., 2006)] could be used as host cells. Nonetheless, for easy and convenient preparation of ABCG2-expressing cells, we here recommend plasmid-based overexpression in non-polarized cells exhibiting high transfection efficiency, such as HEK293 cells (Miyata et al., 2016).

#### Vesicle Transport Assay

Vesicle transport assay is a well-established *in vitro* method employed to quantitatively evaluate ABC transporter function. The presence of ATP-dependent active transport across the cell membrane was directly proved using this method (Ishikawa, 1989). In this assay, ATP-regeneration components—enough amount of creatine phosphate and creatine kinase—are employed to maintain ATP levels in the reaction mixtures during prolonged incubation, and AMP is used as an alternative of ATP for the ATP-deficient controls. After incubation for the transport reaction, the plasma membrane vesicles are washed by filtration and then the intravesicularly accumulated substances are detected. To make this process more convenient and sensitive, radiolabeled or fluorescent substrates are usually used; alternatively, mass spectrometry is used (Toyoda et al., 2016; Takada et al., 2018). With ABCG2, [^14^C]-urate (Miyata et al., 2016; Stiburkova et al., 2016; Higashino et al., 2017) and [^3^H]-estrone sulfate (Suzuki et al., 2003) are well-used radiolabeled substrates that exhibit comparatively lower background signal for the quantitative detection due to their relatively hydrophilic properties. Additionally, non-radiolabeled experiments are conducted by the combined use of hematoporphyrin (a fluorescent ABCG2 substrate) and gel filtration techniques (Tamura et al., 2006).

#### ATPase Assay

Since ABC protein is driven by the free energy of ATP hydrolysis, ATPase activity is recognized as an indicator of the substrate transport. In this assay, the release of inorganic phosphate from ATP coupled with the transport of substrates is estimated using a colorimetric method, such as malachite green procedure (Baykov et al., 1988). This catalytic assay is relatively convenient for estimating the activity of some ABC proteins that prefer lipophilic compounds as their substrates because the non-specifically adsorbed substrates on the vesicles interfere with the measurement of direct transport. Considering that ABCB1 activity has been well studied based on the ATPase assay, this method will be appropriate when investigators would need to compare the inhibitory effects of target compounds on ABCB1 and ABCG2 (Zhang et al., 2016; Guo et al., 2018). Nonetheless, since the ATPase assay does not evaluate the direct transport, regarding ABCG2, we recommend the vesicle transport assay for more precise evaluation.

### Proteoliposome-Based Methods

An artificial lipid membrane system characterized by the reconstitution of purified ABC protein into proteoliposomes (Ambudkar et al., 1998; Jackson et al., 2018) is also a powerful technique. The ABC protein-contained proteoliposomes can be used as an alternative to the plasma membrane vesicles, and their detailed preparation methods are described previously (Geertsma et al., 2008). Additionally, this approach serves as the first choice in the functional studies involving the ABC proteins localized on the organelle membrane (Okamoto et al., 2018).

### Cell-Based Methods

#### Drug Resistance/Accumulation Test

The cells overexpressing MDR machinery show lower sensitivity against its substrate drugs exhibiting cytotoxic or anti-proliferative effects compared to their parent cells, indicating that the ABC protein-expressing cells have higher half maximal effective concentration (EC_50_) values for cytotoxic transporter substrates. In such situations, co-treatment of the transporter inhibitors with the substrate decreases the EC_50_ values, which is depicted as a left-side shift of cell viability curve in the cytotoxic assay. Additionally, if fluorescence transporter substrates are available, flow cytometry analyses addressing their intracellular accumulation will be useful for inhibitor screening (Murakami et al., 2017; Wu et al., 2017).

#### Transcellular System

To investigate the transcellular transport of substances, mono-layer culture of polarized cells expressing target transporter(s) in Transwell^®^ inserts system has been used (Hegedus et al., 2009). For instance, using double-transfected Madin-Darby canine kidney II cells overexpressing both organic anion-transporting polypeptide 1B1 (OATP1B1, a basal uptake transporter) and ABCG2 (an apical efflux transporter), an earlier study has observed an enhanced vectorial transport of [^3^H]estrone sulfate—an ABCG2 substrate that cannot passively penetrate the plasma membrane—from the basal to the apical side (Matsushima et al., 2005). A similar strategy employing ABCG2-expressing polarized cells was used for *in vitro* studies investigating the active secretion of drugs and toxins into milk via ABCG2 (Wassermann et al., 2013; Ito et al., 2015). Of note, in this transcellular system, endogenous transporters and metabolic reactions in the cells may affect apparent transport activities of target transporters.

### *In vivo* Evaluation Methods

#### Xenograft Models

Athymic nude mice models with MDR-xenografts have been used to test whether co-administration of a potential MDR inhibitor with an anti-cancer agent can reverse the MDR phenotype (Tiwari et al., 2013; Zhang et al., 2017, 2018). In such models, the obtained results could be affected by the difference in the origin of the transplanted cancer cells. This concern might be important while xenograft models are studied.

#### Focusing on Pharmacokinetic Characteristics

To examine the *in vivo* effects of different chemicals on the target ABC transporter, its pharmacokinetic role has been focused (Robey et al., 2018). With ABCG2, the pharmacokinetic parameters related to the intestinal absorption or the brain distribution of ABCG2 substrate drugs will be good indicators for ABCG2 activity *in vivo*. For example, pre-dosing of enough quantity of ABCG2 inhibitors increased the bioavailability of sulphasalazine—an ABCG2 substrate—in wild-type mice. This was, however, not observed in *Abcg2*-knockout mice, suggesting the *in vivo* inhibition of Abcg2 (Kusuhara et al., 2012; Miyata et al., 2016). Interestingly, the utility of a combination of brain-specific firefly luciferase transgenic mice and D-luciferin, a chemiluminescent luciferase substrate transported by ABCG2 (Zhang et al., 2007) to investigate the *in vivo* inhibitory effects of test compounds on ABCG2 in the BBB was reported (Bakhsheshian et al., 2016). Further methodological progress will aid evaluation of *in vivo* ABCG2 function.

### Structure-Based *in silico* Approaches

A main approach to abolish MDR is to discover specific inhibitors of the drug-efflux pump. For this purpose, quantitative structure-activity relationship (QSAR) analysis among the series of compounds can serve for the design of lead inhibitors (Nicolle et al., 2009; Ishikawa et al., 2012; Marighetti et al., 2013; Shukla et al., 2014). With ABCG2, since three-dimensional structures of this protein determined by cryo-electron microscopy (EM) were very recently presented (Taylor et al., 2017; Jackson et al., 2018), a deeper understanding of the chemicals–ABCG2 interactions will be achieved as described below.

## History and Recent Update of the Abcg2 Inhibitors

As a MDR machinery in cancer cells and an important drug gatekeeper in tissues like the intestine and the brain, ABCG2 is involved in the efficacy of cancer chemotherapy in patients treated with ABCG2 substrate anti-cancer drugs. To achieve appropriate modulation of ABCG2 by small molecules, the inhibitory potency of various compounds against ABCG2 activity has been extensively evaluated. In this section, we highlight the history and recent update of ABCG2 inhibitors.

### Overview of the History of ABCG2 Inhibitors

The first ABCG2 inhibitor reported was FTC, a mycotoxin produced by *Aspergillus fumigatus* (Rabindran et al., 1998, 2000). The *in vivo* use of FTC was unfortunately precluded due to its neurotoxicity. Among the FTC derivatives, Ko143 was identified as a highly potent ABCG2 inhibitor *in vivo* as it was less neurotoxic than the native FTC and was not overtly toxic to mice (Allen et al., 2002). Cell-based assays showed that the EC_90_ concentrations of Ko143 were 23 nM (Abcg2-mediated mitoxantrone resistance), 5.5 μM (ABCB1-mediated paclitaxel resistance) and >8 μM (ABCC1-mediated etoposide resistance), respectively; these results indicated that Ko143 inhibits ABCG2 stronger than ABCB1 and ABCC1, but is not selective to ABCG2 (Allen et al., 2002). Furthermore, from a series of ABCB1 inhibitors, some ABCG2 inhibitors, such as elacridar (GF120918) (Allen et al., 1999; Kruijtzer et al., 2002) and tariquidar (XR9576) (Robey et al., 2004) are frequently used in basic research as well as Ko143.

To date, the molecular bases relating to the chemical inhibition of ABCG2 are not fully understood. The cryo-EM structures of ABCG2 (Taylor et al., 2017) and ABCG2 bound to Ko143 derivatives or tariquidar (Jackson et al., 2018) will be an important to address this issue. Besides, another group of researchers has revealed the structural characteristics of ABCG2 protein critical for its function based on a molecular modeling approach combined with biochemical characterizations of ABCG2 mutants (Khunweeraphong et al., 2017). Previous studies employing the QSAR approaches predicted some structural requirements of compounds for interacting with ABCG2 as an inhibitor (Ishikawa et al., 2012; Mao and Unadkat, 2015); not being true for all ABCG2 inhibitors, the representative features are lipophilicity, planner structure, and amine bonded to one carbon of a heterocyclic ring. Furthermore, a virtual screening strategy employing a ligand-based *in silico* classification model to predict the inhibitory potential of drugs toward ABCG2 presented some favorable outcomes (Montanari et al., 2017). Integration of these findings will contribute to providing a basis for the design of new ABCG2 inhibitors.

Hitherto, many studies focusing on the chemicals–ABCG2 interactions identified a large number of ABCG2 inhibitors with diverse chemical structures (Mao and Unadkat, 2015; Wiese, 2015; Pena-Solorzano et al., 2017; Silbermann et al., 2019). An expanding list of the ABCG2 inhibitors, which include ABCG2 substrates (competitive inhibitors), contains such drugs on the market as TKIs and anti-HIV drugs [some of them are often ABCG2 substrates (Polgar et al., 2008; Mao and Unadkat, 2015)] and such dietary phytochemicals as flavonoids and rotenoids. Nevertheless, to the best of our knowledge, the clinical use of such chemicals for ABCG2 inhibition has not yet been achieved probably due to concerns on safety and/or *in vivo* efficacy of them, which may have been common reasons responsible for the failure of the clinical development of ABCG2 inhibitors. Recent studies importantly showed that two potential anti-cancer compounds under clinical development could competitively inhibit both ABCG2 and ABCB1 (Ji et al., 2018a,b).

### Febuxostat, a Highly Potent ABCG2 Inhibitor Applicable in Clinical Situations

**FIGURE 2:**
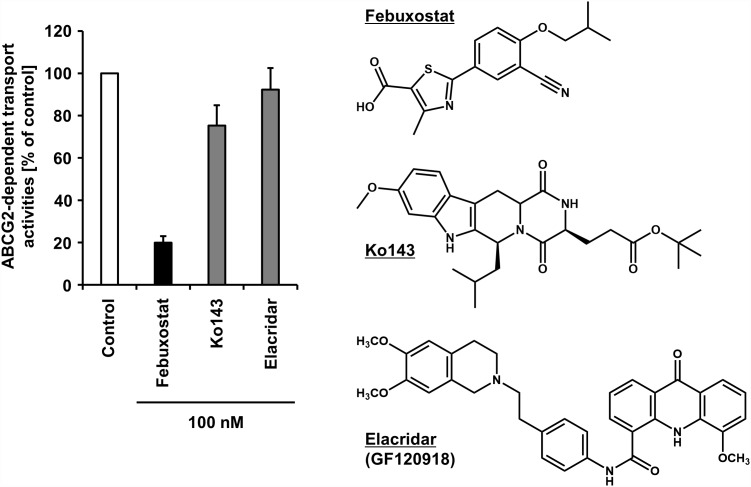
Inhibitory effect of febuxostat against ABCG2 is stronger than that of Ko143 and elacridar, two well-used ABCG2 inhibitors. Febuxostat is known as an oral hypouricemic agent inhibiting xanthine oxidoreductase, a key enzyme for uric acid production. **(Left)** Effect of each compound on the transport activity of ABCG2 are shown. Data from our previous study under CC BY license (Miyata et al., 2016) are shown graphically, in which in the absence (vehicle control) or presence of 100 nM of each compound, the ATP-dependent urate transport activities of ABCG2 were measured using the vesicle transport assay. Data are expressed as the mean ± SD, *n* = 3. Of note, the half-maximal inhibitory concentration (IC_50_) of febuxostat against the urate transport activity of ABCG2 was 27 nM in the previous study. **(Right)** Chemical structures of each compound are depicted.

Regarding the difficulty in the clinical applications of existing ABCG2 inhibitors, our recent study may open up further avenues, in which febuxostat—an approved agent for hyperuricemia globally used in clinical situations—was serendipitously identified as a strong ABCG2 inhibitor both *in vitro* and *in vivo* (Miyata et al., 2016). Using the vesicle transport assay, we revealed that febuxostat inhibits ABCG2 more strongly than Ko143 and elacridar (Figure 2). This indicates that febuxostat has a superior safety profile and better inhibitory ability against ABCG2 compared to these two compounds. Moreover, the study demonstrated that the IC_50_ of febuxostat against urate transport activity of ABCG2 (0.027 μM) was lower than its maximum plasma unbound concentrations reported in humans (0.09 μM), suggesting that febuxostat might inhibit human ABCG2 at a clinically used dose. Thus, febuxostat can be a promising candidate as a potential ABCG2 inhibitor in humans. The structural characteristics and molecular mechanisms of febuxostat as an ABCG2 inhibitor remain to be elucidated, as well as the effects of febuxostat on the function of other ABC transporters, including ABCB1.

Febuxostat will be used in cancer chemotherapy more frequently because recently this drug has been approved in Europe and Japan for the prophylaxis of TLS. TLS is a potentially life-threatening condition caused by an abrupt release of intracellular metabolites after tumor cell lysis in cancer patients on chemotherapy (Alakel et al., 2017). It is the most common treatment-related emergency in patients with hematologic malignancies and characterized by metabolic abnormalities including hyperuricemia that triggers several mechanisms resulting in acute kidney injury. Appropriate control of serum uric acid is therefore important in the prevention of TLS. Recent studies demonstrated that febuxostat—an oral hypouricemic agent—can successfully prevent TLS in cancer patients (Spina et al., 2015; Tamura et al., 2016). In such situations, since the patients will be treated with febuxostat before and during chemotherapy, there would be drug-drug interactions between febuxostat and ABCG2 substrate anti-cancer agents.

Importantly, ABCG2 is reportedly expressed on the malignant hematopoietic and the lymphoid cells frequently; its expression in several types of human leukemia has been investigated (Jordanides et al., 2006; Eechoute et al., 2011; Stacy et al., 2013). Considering that initiating cells in CML arises from a multipotent hematopoietic stem cell (HSC), together with high expression of ABCG2 in human HSCs (Zhou et al., 2001; Scharenberg et al., 2002), overexpression of ABCG2 has been considered to confer drug resistance ability to the CML stem cell population (Brendel et al., 2007). However, the clinical relevance of ABCG2 inhibition in CML patients from the view point of reversing MDR remains to be clarified. Since several TKIs used for CML are ABCG2 substrates (Hira and Terada, 2018), the combination use of febuxostat may be a future research topic.

Additionally, ABCG2 expressed in tumor cells is reportedly involved in the efflux of photosensitizers in 5-aminolevulinic acid-based photodynamic therapy (Ishikawa et al., 2015). Hence, febuxostat might enhance the efficacy of this minimally invasive modality for treating solid cancers by accumulating photosensitizers in the target cells. The details are discussed in our previous report (Miyata et al., 2016).

## Conclusion and Perspective

Here, we summarized some key experimental systems that will continuously contribute to generating novel ABCG2 inhibitors and described an overview of the current update of ABCG2 inhibitors. Among them, febuxostat will be one of the most promising candidates for clinical use. Considering that dysfunctional *ABCG2* genotypes, which are summarized in a recent review (Heyes et al., 2018), alter the pharmacokinetic characteristics of ABCG2 substrate drugs such as several TKIs (Hira and Terada, 2018) and rosuvastatin (Keskitalo et al., 2009), ABCG2 inhibitors will also exert similar effects in humans. As a beneficial application of this clinical possibility, we have proposed a novel concept named febuxostat-boosted therapy (Miyata et al., 2016), in which febuxostat is expected to enhance the bioavailability of ABCG2 substrate drugs. For a similar purpose, ritonavir and cobicistat are used as pharmacokinetic boosters inhibiting cytochrome P450 3A4, a major pathway of drug metabolism, to increase the plasma concentrations of certain drugs (Shah et al., 2013). No pharmacokinetic enhancer targeting transporter proteins has been, however, successfully evaluated in clinical trials. In this context, the potential benefits of the febuxostat-boosted therapy should be validated in the near future. Furthermore, this concept could also be applied to enhance the BBB penetration of ABCG2 substrate drugs for brain cancer chemotherapy. Despite the potential risks of adverse events in the combination therapy, further clinical studies to elucidate whether febuxostat is beneficial in enhancing the efficacy of pharmacotherapy via ABCG2 inhibition are warranted.

## Author Contributions

YT researched the data for the manuscript, provided substantial contributions to discussion of its content, and wrote the manuscript. TT contributed to the discussion and the writing of the manuscript. HS critiqued the manuscript and provided intellectual inputs. All the authors reviewed and edited the manuscript before submission and have made final approval of the manuscript.

## Conflict of Interest Statement

TT and HS have a patent pending. The remaining author declares that the research was conducted in the absence of any commercial or financial relationships that could be construed as a potential conflict of interest.

## Abbreviations

ABC: ATP-binding cassette
BBB: blood–brain barrier
CML: chronic myeloid leukemia
EC_50_: half-maximal effective concentration
FTC: fumitremorgin C
IC_50_: half-maximal inhibitory concentration
MDR: multidrug resistance
TKI: tyrosine kinase inhibitor
TLS: tumor lysis syndrome
